# SIRPγ-CD47 Interaction Positively Regulates the Activation of Human T Cells in Situation of Chronic Stimulation

**DOI:** 10.3389/fimmu.2021.732530

**Published:** 2021-12-01

**Authors:** Safa Dehmani, Véronique Nerrière-Daguin, Mélanie Néel, Nathan Elain-Duret, Jean-Marie Heslan, Lyssia Belarif, Caroline Mary, Virginie Thepenier, Kevin Biteau, Nicolas Poirier, Gilles Blancho, Fabienne Haspot

**Affiliations:** ^1^ OSE Immunotherapeutics, Nantes, France; ^2^ Nantes Université, Inserm, Centre de Recherche en Transplantation et Immunologie, Unité Mixte de Recherche (UMR) 1064, Institut de Transplantation Urologie-Néphrologie (ITUN), Nantes, France

**Keywords:** graft-*versus*-host disease, SIRPA, chronic stimulation, T cell, CD47, SIRPG

## Abstract

A numerous number of positive and negative signals *via* various molecules modulate T-cell activation. Within the various transmembrane proteins, SIRPγ is of interest since it is not expressed in rodents. SIRPγ interaction with CD47 is reevaluated in this study. Indeed, we show that the anti-SIRPγ mAb clone LSB2.20 previously used by others has not been appropriately characterized. We reveal that the anti-SIRPα clone KWAR23 is a Pan anti-SIRP mAb which efficiently blocks SIRPα and SIRPγ interactions with CD47. We show that SIRPγ expression on T cells varies with their differentiation and while being expressed on Tregs, is not implicated in their suppressive functions. SIRPγ spatial reorganization at the immune synapse is independent of its interaction with CD47. *In vitro* SIRPα-γ/CD47 blockade with KWAR23 impairs IFN-γ secretion by chronically activated T cells. *In vivo* in a xeno-GvHD model in NSG mice, the SIRPγ/CD47 blockade with the KWAR23 significantly delays the onset of the xeno-GvHD and deeply impairs human chimerism. In conclusion, we have shown that T-cell interaction with CD47 is of importance notably in chronic stimulation.

## Introduction

T cells are adaptive immune cells that are educated in the thymus to defend the organism and eliminate pathogens efficiently by recognizing the MHC-antigen complex *via* their TCR. Mature T cells express a variety of receptors which engagement will stabilize, enhance, or decrease their activation upon antigen recognition. In this study, we focused on SIRPγ, a T-cell restricted surface molecule from the signal-regulatory protein (SIRP) family. SIRP family comprise transmembrane glycoproteins expressed on immune cells and central nervous system (1–3). Three main members have been described: SIRPα, SIRPβ, and SIRPγ with homologous extracellular immunoglobulin (Ig)-like domains but distinct transmembrane and intracytoplasmic domains (4–8). Briefly, SIRPα (CD172a) is expressed on hematopoietic progenitors, myeloid cells, dendritic cells, NK cells, and neurons (4, 9). SIRPα cytoplasmic tail contains immunoreceptor tyrosine-based inhibition motifs (ITIMs) (10) resulting in inhibitory signals. SIRPα ligates CD47, an ubiquitously expressed molecule, and this interaction is responsible of the “don’t eat me signal” which leads to the inhibition of phagocytosis by myeloid cells (11). SIRPβ (CD172b) associates with DAP12 (12, 13) which contains an immunoreceptor tyrosine-based activation motif (ITAM). Yet, ligand of SIRPβ is still unknown. Finally, SIRPγ (CD172g) has been described recently (14) probably because the gene of SIRPγ is absent in rodents and found only in humans and primates (15). SIRPγ has a very short intracytoplasmic tail of 4-amino acids (aa) uncapable of transducing signals on its own. SIRPγ also interacts with CD47 as SIRPα (14–16), but the SIRPα/CD47 affinity (Kd 2 µM) is 10 times stronger than the one of SIRPγ/CD47 (Kd 23 µM) (17).

It has been shown that the interaction between SIRPγ and CD47 plays a key role in T-cell transendothelial migration under shear flow conditions (17) in cell-cell adhesion and promotes antigen-specific T-cell proliferation and costimulation (16). However, these observations rely on the use of the anti-SIRPγ clone LSB2.20 which we report in this study as not being able to efficiently block the SIRPγ/CD47 interaction at the concentration tested. Thus, we reevaluate the impact of SIRPγ in human T-cell biology using a different anti-SIRP monoclonal antibody (clone KWAR23), which we characterize here as capable to efficiently block SIRPγ and SIRPα interactions with CD47. We show that SIRPγ expression varies on CD4 and CD8 T-cell subpopulations and, while being expressed by regulatory T cells (Tregs), we demonstrate that SIRPγ/CD47 interaction is not implicated in their suppressive function. Upon antigen presenting cell (APC) recognition, SIRPγ clusters at the immune synapse independently of its ligation to CD47 on the APC. IFN-γ secretion by chronically activated T cells is impaired by SIRPγ/CD47 interaction blockade *in vitro*. Finally, when used *in vivo* in a T-cell-dependent xeno-GvHD model mediated by the injection of human PBMC in NSG-recipient mice, the anti-SIRPα-γ mAb KWAR23 impairs human cell engraftment, leading to a delayed GvHD onset. Importantly, all CD4 and CD8 T-cell compartments were compromised in KWAR23-treated mice. In conclusion, we have shown that T-cell interaction with CD47 is of importance notably in chronic stimulation.

## Materials and Methods

### Reagents

All the reagents used in the study are listed in the **Table 1**.

**Table 1:** Key resource table.

Reagent type	Designation	Source	Reference	Host and subclass	Clone name
Antibody	Antihuman CD3	BD Biosciences	557943, 555332, 583725	Mouse IgG1, κ	UCHT1
Antibody	Antihuman CD4	BD Biosciences	562424, 560158, 55346, 560768	Mouse IgG1, κ	RPA-T4
Antibody	Antihuman CD4	BD Biosciences	557852	Mouse IgG1, κ	SK3
Antibody	Antihuman CD8	BD Biosciences	564116	Mouse IgG1, κ	SK1
Antibody	Antihuman CD14	BD Biosciences	558121	Mouse IgG_2a_, κ	M5E2
Antibody	Antihuman CD45RA	BD Biosciences	550855, 563870, 550994	Mouse IgG2b, κ	HI100
Antibody	Antihuman CD45	BD Biosciences	563792	Mouse IgG1, κ	HI30
Antibody	Antihuman CD27	BD Biosciences	564301	Mouse IgG_1_	L128
Antibody	Antihuman CD279 (PD1)	BioLegend, San Diego, CA, USA	329918	Mouse IgG1, κ	EH12.2H7
Antibody	Antihuman CCR7	BD Biosciences	557648	Mouse IgG2a, κ	3D12
Antibody	Antihuman CD95	BD Biosciences	559773	Mouse IgG1, κ	DX2
Antibody	Antihuman CD28	BD Biosciences	562296	Mouse IgG1, κ	CD28.2
Antibody	Antihuman CD47	BD Biosciences	560371	Mouse IgG1, κ	B6H12
Antibody	Antihuman CD25	BD Biosciences	555431,563701	Mouse IgG1, κ	M-A251
Antibody	Antihuman CD127	BD Biosciences	557938, 562662	Mouse IgG1, κ	HIL-7R-M21
Antibody	Antihuman CD127	BioLegend	351318	Mouse IgG1, κ	A019D5
Antibody	Antihuman SIRPγ	BioLegend	336606	Mouse IgG1, κ	LSB2.20
Antibody	Antihuman CD47	R&D Systems, Minneapolis, MN, USA	FAB4670G	Mouse IgG1	472603
Antibody	Antihuman FoxP3	eBioscience, San Diego, CA, USA	45-4776-42	Rat IgG2a, κ	PCH101
Antibody	Antimouse CD45	BD Biosciences	550994	Rat IgG_2b_, κ	30-F11
Antibody	Antimouse SIRPα	BD Biosciences	740282	Rat IgG1, κ	P84
Antibody	Antimouse CD11b	BD Biosciences	552850	Rat IgG2b, κ	M1/70
Antibody	Antihuman Fc	BioLegend	410708, 409304	Rat IgG2a, κ	M1310G05
Antibody	Antihuman CD3	In house	Hybridoma	Mouse IgG2a, κ	OKT3
Antibody	Pan antihuman SIRP	Produced by OSE Immunotherapeuthics	Ring et al. (18) WO2015138600A2	Chimeric human-IgG4	Kwar23
Antibody	Antihuman SIRPα	OSE Immunotherapeuthics		Mouse IgG	18D5
Antibody	Antihuman CD47				B6H12
Antibody	Antihuman IgG (H+L)	Jackson Immunoresearch, West Grove, PA, USA	709-005-098	Donkey	
Antibody	Antihuman IgG	Jackson Immunoresearch	709-035-149	Donkey	
Antibody	Antihuman IgG Fc	BioLegend	409303		
Recombinant protein	hSIRPα	SinoBiological	11612-H08H	Human	
Recombinant protein	SIRPγ	SinoBiological	11828-H08H	Human	
Recombinant protein	CD47	SinoBiological	12283-H02H	Human	
Recombinant protein	CD47	AcroBiosystems, Newark, DE, USA	CD7-H82F6	Human	
Commercial kit	Pan T-cell isolation kit	Miltenyi, Bergisch Gladbach, Germany	130-096-535		
Toxin	SEE	Toxin Technology, Inc. ET404, Sarasota, FL, USA			
Reagent	Human Fc receptor-binding inhibitor	BD Pharmingen, San Diego, CA, USA	564220		
Reagent	Human IFNg ELISA set	BD Biosciences	555142		
Reagent	Alamar Blue	Invitrogen, Waltham, MA, USA	DAL1100		
Reagent	Streptavidin peroxidase	Jackson Immunoresearch	016-030-084		
Reagent	Cell Proliferation Dye eFluor™ 450	eBioscience™	65-0842-85		
Reagent	Cell Proliferation Dye eFluor™ 670	eBioscience™	65-0840-85		
Cell tracker	Cell Tracker™ Green CMFDA Dye	ThermoFisher, Waltham, MA, USA	C7025		
Reagent	Poly-l-lysine	Sigma	P4832		
Microslides	IBIDI 8 wells	IBIDI	80826		

### Cells

Human peripheral blood mononuclear cells (PBMC) were isolated from fresh cytapheresis ring from healthy donors obtained from the Etablissement Français du Sang (Nantes, France). Written informed consent was provided according to institutional guidelines. Human T cells were isolated by negative selection with the Miltenyi’s Pan T-cell isolation kit according to the manufacturer’s protocol. Jurkat T cells (ATCC, clone E6-1) were cultured at 10^5^ cells/ml in complete RPMI medium (10% GE Healthcare HyClone™ Fetal Bovine Serum (Chicago, IL, USA), 1% penicillin-streptomycin Gibco^®^ (Waltham, MA, USA), 1% glutamine, 1% sodium pyruvate Gibco^®^, 1% Hepes Gibco^®^, and 1% MEM NEAA Gibco^®^) at 37°C, 5% CO_2_.

### Development of KO and KI Jurkat

The GeneArt CRISPR Nuclease Vector kit (Life Technologies, Carlsbad, CA, USA) was used. Guide RNAs for SIRPγ and CD47 were selected thanks to the CRISPOR website. The following were the sRNA selected: sgRNA anti-SIRPg #1 TTCCCGTGGGACCCGTCCTG *TGG*, sgRNA anti-SIRPg #2 TAGTATGTGCCGACATCTGC *TGG*, sgRNA anti-CD47#1 CTTGTTTAGAGCTCCATCAA *AGG*, and sgRNA anti-CD47#2 TCCATGCTTTGTTACTAATA *TGG*; PAM sequence is indicated in italic. Briefly, Jurkat cells suspended in Opti-MEM (Gibco) medium at 10^6^ cells/ml were electroporated with a NEPA21 (Nepalgene) and placed immediately in warm complete RPMI medium in six-well plates and incubated at 37°C, 5% CO_2_. After amplification, cells were stained using anti-SIRPγ mAb and/or anti-CD47 mAb for FACS sort with the ARIA II (BD Biosciences, Franklin Lakes, NJ, USA) depending on the nonexpression of the corresponding epitopes: SIRPγ, CD47. Sorted cells were amplified for ulterior use. Genomic DNAs were extracted, and PCR amplified with the following primers SIRPγ F1 5′-GCCTCAGTGCCCTCAATTGT-3′; SIRPγ R1 5′-GGGATGAGGGAGGTCCATGT-3′; SIRPγ F2 5′-TGTGCACCCAGTCACTGAATA-3′; SIRPγ R2 5′-GGGGTGACAACAGGTCTTGA-3′; CD47 F1 5′-CTTCAAAGCTTCCAAAGCCAGA-3′; CD47 R1 5′-AAGAGGATCAGGTTGCACCA-3′; CD47 F2 5′-ACTACACCTGCATGTTCCAA-3′; and CD47 R2 5′-CAGGTTGCACCAGGACAAAT-3′.

Jurkat SIRPγ-KO cells were then transduced with lentivirus encoding OST-SIRPγ. SIRPγ expression on transduced cells was higher than in their WT counterpart. SIRPγ^high^ cells were FACS sorted and amplified for ulterior use.

### ELISA-Binding Assay

For human SIRPα-binding assay, recombinant human SIRPα was immobilized on plastic at 0.5 µg/ml (SIRPα) in carbonate buffer (pH 9.2). After saturation, purified antibodies were added in range (from initial concentration at 10 µg/ml) to measure binding. After incubation and washing, peroxidase-labeled donkey antihuman IgG was added and revealed by conventional methods.

For human SIRPγ-binding assay, purified antibodies were captured in range (from initial concentration at 5 µg/ml) with a coated donkey antihuman IgG (H+L) immobilized at 2 µg/ml in borate buffer. After washing, biotinylated SIRPγ (#11828-H08H, SinoBiological, Beijing, China, biotinylation performed by OSE Immunotherapeutics, Nantes, France) was added at 1 µg/ml and detected by streptavidin peroxidase, then revealed by conventional methods.

### Human SIRPγ-Binding Assay on Human Lymphocyte and Monocyte by Flow Cytometry

PBMC, isolated by Ficoll from blood sample, were incubated with human Fc receptor-binding inhibitor diluted at 1/50. Then, antibodies were incubated for 30 min at 4°C and washed before being stained 30 min at 4°C with PE-labeled antihuman IgG Fc and with a mix of 50-fold diluted antibodies to determine cell subpopulations (CD3^+^ for T lymphocyte and CD14^+^ for monocyte). Samples were analyzed on CytoFlex cytofluorometer (Beckman Coulter France, Villepinte, France) after gating on different subpopulations.

### Human SIRPγ and Human SIRPα Antagonist Activity Measured by ELISA

For antagonist activity, enzyme-linked immunosorbent assay (ELISA) assay, recombinant human SIRPγ, or recombinant human SIRPα were immobilized on plastic at respectively 2 µg/ml (SIRPγ) and 0.5 µg/ml (SIRPα) in carbonate buffer (pH 9.2). During saturation, biotinylated human CD47 were preincubated at a unique concentration (final concentration at 3 µg/ml) with purified antibodies in range at room temperature for 15 min. Preincubated (CD47-biot/purified antibodies) mixes were then incubated on immobilized SIRPγ (O/N at room temperature) or on immobilized SIRPα (for 2 h at 37°C). After incubation and washing, streptavidin-peroxidase was added and revealed by conventional methods.

### Flow Cytometry Assays

PBMC (10^5^ per well) were suspended in 200 μl of FACS buffer (phosphate-buffered saline 1×, 1% bovine serum albumin, Sigma-Aldrich, 0.1% Azide Sigma-Aldrich, St. Louis, MO, USA) and plated in a V-bottom 96-well plate prior to being centrifuged at 2,500 rpm for 1 min. For the extracellular staining, cells were labeled with a mix of antibodies for 30 min on ice. For the intracellular labeling, BD Cytofix/Cytoperm kit was used according to the manufacturer’s protocol with recommended volume of intracellular antibodies. Cells were washed twice before analysis on the LSRII (BD Biosciences). After the acquisition, data were analyzed using FlowJo software.

### Coculture of Tregs and Effector T Cells

Purified T cells were stained with anti-CD4, anti-CD25, and anti-CD127 for 30 min at 4°C in the dark, cells were washed, and DAPI was added to discriminate dead cells. Cells were then sorted using the ARIA II (BD Biosciences). Tregs and effector T cells were further stained with two different cell proliferation dyes (CPD) for 10 min at 37°C and cultured either alone (control conditions) or together, in U-96-well costar plates previously coated or not with anti-CD3 (clone OKT3) at 2 µg/ml. When indicated, soluble anti-SIRPα/γ (KWAR23) antibody was added at 10 µg. After 5 days in culture, T-cell proliferation was assessed by flow cytometry using the LSRII (BD Biosciences).

### T-Cell Activation

T cells were stained with CPD prior to be activated in plates coated with 0.5 µg/ml of anti-CD3 (OKT3) and 2–3 µg/ml of soluble anti-CD28.2. Cells were incubated 3 or 5 days at 37°C, 5% CO_2_, then assessed by flow cytometry. In some experiments, T cells were stimulated with 0.5–1 µg/ml of coated OKT3 with or without coated CD47-Ig (10 µg/ml).

### Chronically Activated T Cells

PBMC at 7.5 × 10^6^ were stimulated in complete medium three times in six-well plates coated with 3 µg/ml of anti-CD3 (OKT3) and 3 µg/ml of soluble anti-CD28.2. Each stimulation is consist of a 48–72-h culture in six-well plates coated with 3 µg/ml of anti-CD3 (OKT3) and 3 µg/ml of soluble anti-CD28.2. Each time, cells were washed and counted prior to be restimulated. Chronically stimulated PBMC were then cultured for 48 h in plates coated with 0.5 µg/ml of anti-CD3 (OKT3) and 10 µg/ml of human CD47-Fc recombinant protein in the presence or not of mAbs targeting the SIRP/CD47 interactions. Supernatants were collected after 48 h of stimulation with OKT3 and CD47-Fc to measure IFN-γ secretion, and cell fitness was analyzed by Alamar Blue detection at 530 nm after 5 days stimulation.

### Confocal Microscopy

Jurkat, resting, or activated T cells were stained for 30 min at 4°C in the dark with an anti-SIRPγ (LSB2.20) and an anti-CD47 mAb. Cells were fixed with PFA at 4% for 10 min at room temperature (RT), permeabilized with PBS + 0.1% Triton for 5 min at RT, and further stained with DAPI for 5 min at RT in the dark. ProLong was added to the cells and the solution carefully place between a glass slide and a 12-µM round coverslip and left for at least 48 h polymerization before being analyzed with confocal microscopy (Nikon Super Resolution SIM Confocal A1) using the NIS software. The images obtained were analyzed using ImageJ software.

SIRPγ and CD47 colocalization was analyzed using ImageJ software. Briefly, a region of interest (ROI) was drawn on the whole cell surface and analyzed with coloc2 plugin.

### Time-Lapse Microscopy

IBIDI (Gräfelfing, Germany) 8-well slides were coated with poly-l-lysine at 0.001%. Raji cells were primed with 10 ng/ml of SEE for 30 min at 37°C, and WT or SIRPγ^high^ Jurkat cells were stained with a cell tracker green for 15 min at 37°C, washed thoroughly then stained with an anti-SIRPγ mAb (clone LSB2.20) for 30 min at 4°C. Cells were then washed, and Raji and Jurkat cells were deposited in the IBIDI slide. Once the cells are stabilized, the time lapse video starts. To assess the role of SIRPγ in the synapse formation, SIRPγ/CD47 interaction was blocked on SIRPγ^high^ cells for 15 min at 37°C with the KWAR23 mAb at 10 µg/ml, then mixed with Raji for 15 min prior to be plated in IBIDI wells. SIRPγ polarization into the synapse was analyzed using ImageJ software. Briefly, a ROI was drawn at the cell/cell contact between Jurkat and Raji and the intensity of pixels for SIRPγ channel was analyzed. The same ROI was then moved at the opposite side of the synapse to measure the intensity of pixels for SIRPγ channel. For control condition, cells were divided randomly in half and the intensity of pixels for SIRPγ channel was measured on the two parts of the cells. The size of the ROI does not influence the intensity of pixels measured.

### Animals

We used a xeno-GvHD model of immunodeficient mice to assess the role of SIRPγ in GvHD occurrence. NSG mice were purchased from the Jackson Laboratory (Bar Harbor, ME) and bred by the LabEx IGO humanized rodent platform. Mice (males and females) were used between 8 and 12 weeks old. All animals were housed under specific pathogen-free conditions, according to institutional guidelines. NSG mice were irradiated at 1.5 Gy on Day 0 prior to an i.v. injection of 10^7^ human PBMC from healthy volunteers on Day 0. Mice were treated 2 times/week with PBS or anti-SIRPα-γ (KWAR23 mAb) at 5 mg/kg from D1 to D42. Human cell engraftment and weight lost were monitored during the experiment. If losing more than 20% of its maximum body weight, the mice were sacrificed. Human chimerism analyzed by flow cytometry is calculated as follows: (% of hCD45)/(% of hCD45 + % of mCD45) * 100. The relative number of CD4/CD8 T cells in the spleen is calculated as follows: (% human chimerism in spleen) * (% human CD4/CD8 in spleen). This study was carried out according to authorization from the French Ministry of Research, APAFIS # 4678. Intraperitoneal residual macrophages were collected as previously described in {Ray:2010cp}. Fc block was added to the cells following manufacture recommendation prior the incubation with the antibodies.

## Results

### The KWAR23 Is a Pan Anti-SIRP mAb, Which Efficiently Inhibits SIRPγ-CD47 Interaction

**Figure 1:**
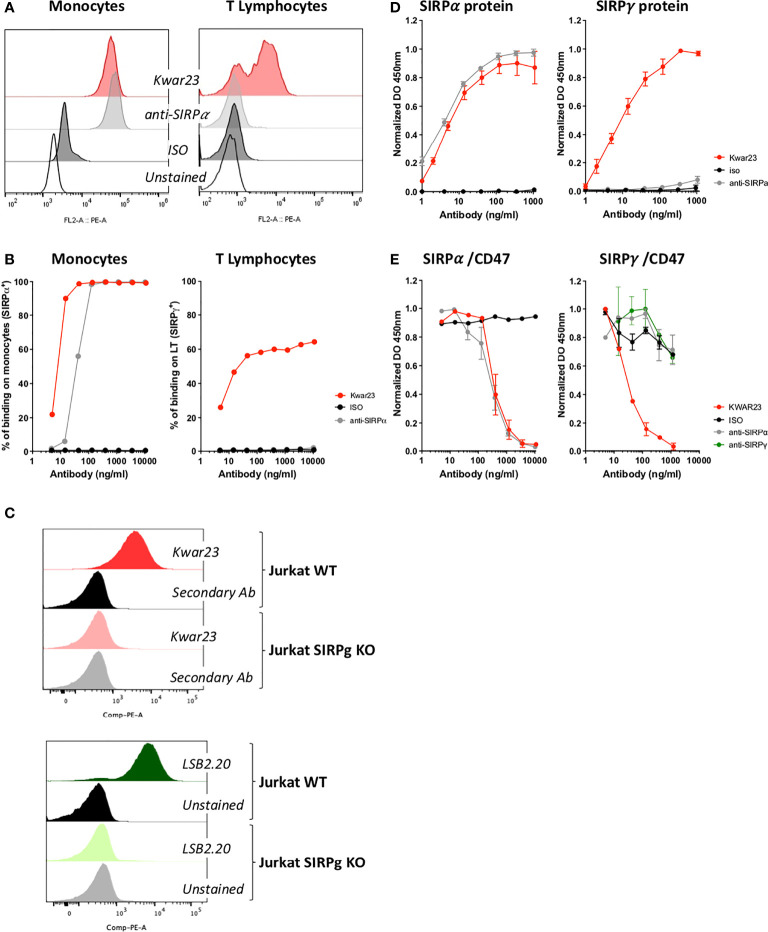
The KWAR23 is a Pan SIRP mAb which impairs SIRPα and SIRPγ interactions with CD47. **(A)** Human monocytes and T cells were labeled with KWAR23, anti-SIRPα mAb (18D5) or an isotype control (ISO) all at 10,000 ng/ml and further analyzed by FACS. **(B)** The percentage of stained cells (monocytes and T cells) is presented regarding the concentration of mAb (*n* = 1). **(C)** KWAR23-binding specificity towards SIRPγ was evaluated by FACS staining on wild-type (WT) and SIRPγ−KO Jurkat cells (upper overlay). LSB2.20 (anti-SIRPγ)KWAR23 recognized staining was used as control (lower overlay). **(D)** Specificity of mAb used in **(A)** and **(B)** were assessed by ELISA against recombinant SIRPα and SIRPγ proteins. Binding activity is presented as normalized absorbance (DO) at 450 nm in function of concentration of mAb (ng/ml), *n* = 2–4 individual experiments (mean ± SEM). **(E)** Antagonist activity of the KWAR23, the anti-SIRPα (18D5), and the anti-SIRPγ (LSB2.20) mAbs to SIRPα/CD47 (left panel) and SIRPγ/CD47 (right panel) binding was measured by ELISA and presented as normalized absorbance (DO) at 450 nm in function of mAb added (ng/ml). *n* = 3 individual experiments (mean ± SEM).

KWAR23 mAb is a recombinant antibody which has been selected after immunization with a SIRPα recombinant molecule by Ring and collaborators (18). Given the homology of 79% when comparing the extracellular portions of the SIRPα, SIRPγ, and SIRPβ (15), we hypothesized that KWAR23 could also recognize other SIRP protein family members. We directly addressed this question by labelling human cells which are known to either uniquely express SIRPα, i.e., monocytes or described to express SIRPγ, i.e., T lymphocytes. KWAR23 efficiently binds 100% of monocytes when the concentration used is sufficient as does the anti-SIRPα-specific mAb (**Figures 1A, B**, left panels); similar results were obtained when U937 (SIRPα^+^ cell line) were stained (data not shown). Interestingly, increased concentration of KWAR23 resulted in T-cell staining which reached a plateau when 60% of human T cells were stained. No binding was seen with the anti-SIRPα-specific mAb (clone 18D5), suggesting that KWAR23 recognizes a molecule other than SIRPα, presumably SIRPγ on T cells (**Figures 1A, B**, right panels). Furthermore, Jurkat WT and Jurkat SIRPγ cells were stained with KWAR23 and LSB2.20, a specific anti-SIRPγ, developed by Piccio and colleagues (16). While both Abs similarly stained Jurkat WT cells, no staining was observed on Jurkat SIRPγKO cells with either Ab, identifying SIRPγ as the sole KWAR23 epitope on Jurkat cells (**Figure 1C**). To confirm these results, we setup two ELISA in which recombinant SIRP molecules were used. The anti-SIRPα-specific mAb recognized SIRPα but not SIRPγ-recombinant protein, while the KWAR23 recognized SIRPα, SIRPγ, and even SIRPβ (**Figure 1D** and data not shown for SIRPβ). Thus, KWAR23 appears as a pan anti-SIRP antibody.

KWAR23 has been selected by Ring and collaborators for its ability to specifically block the SIRPα/CD47 interaction as does the anti-SIRPα Ab (19) (**Figure 1E**, left panel). LSB2.20 inhibits the SIRPγ/CD47 (16). Thus, we evaluated LSB2.20, KWAR23, and the anti-SIRPα for their ability to interfere with SIRPγ/CD47 binding. Surprisingly, the LSB2.20 did not efficiently, block the SIRPγ/CD47 interaction at the concentration tested, neither did the anti-SIRPα. On the contrary, KWAR23 was found to efficiently abrogate SIRPγ/CD47 interaction (**Figure 1E**, right panel).

Thus, we redefined the KWAR23 as a Pan anti-SIRP antibody able to specifically block SIRPα-γ/CD47 interactions.

### SIRPγ Expression Varies on T-Cell Populations and While Being Highly Expressed on Treg, Is Not Implicated in Their Suppressive Functions

**Figure 2:**
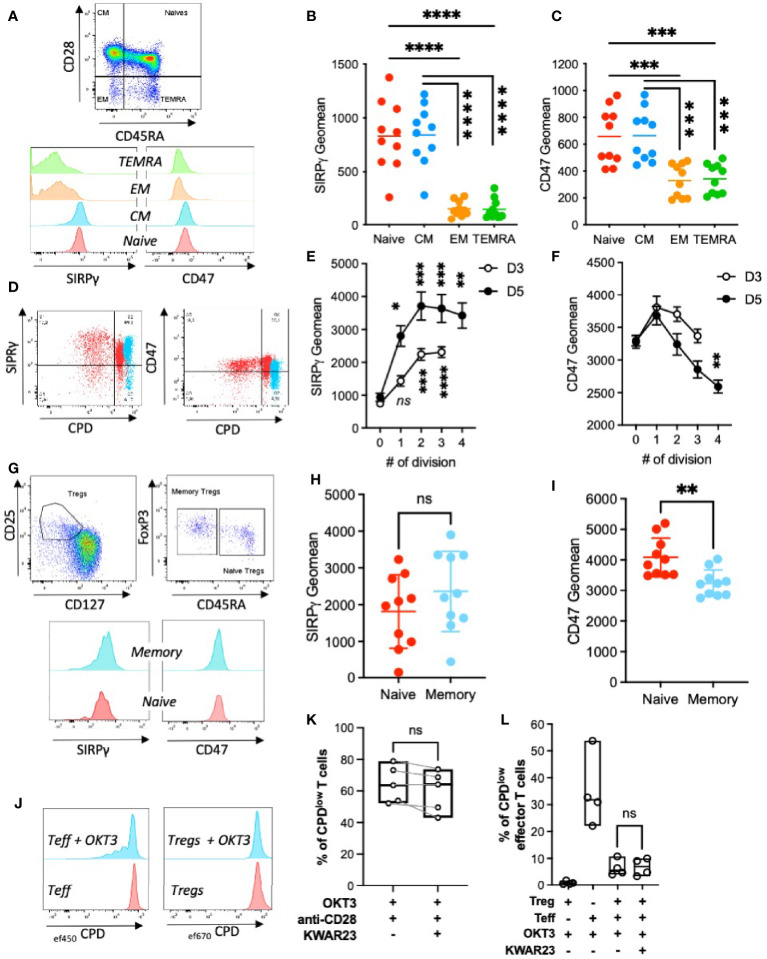
SIRPγ expression varies on T-cell subpopulations, upon T-cell activation and is not implicated in Tregs function. **(A)** T-cell subpopulations gating strategy and their SIRPγ and CD47 expressions. **(B)** Geometric mean of SIRPγ and **(C)** CD47 on T-cell subpopulations; each symbol represents independent HV. Statistical analysis was performed by a Friedman test followed by a Dunn’s multiple comparison test. **(D**–**F)** SIRPγ and CD47 expressions were analyzed after T-cell stimulation with coated anti-CD3 and anti-CD28 mAbs. **(D)** Overlay allowing the comparison of SIRPγ (left) and CD47 (right) expression on unstimulated purified T cells (i.e., Day 0 in blue) *vs*. proliferating CPD^low^ T cells (i.e., Day 5 in red). **(E)** Geometric mean (± SEM) of SIRPγ or **(F)** CD47 on T cells as a function of their number of divisions, *n* = 10 HV analyzed in two independent experiments. Kruskal-Wallis test was used. **(G)** Treg gating strategy based on CD25 and CD127 expression is represented as dot plots; representative histograms of SIRPγ and CD47 staining on Tregs subpopulations are shown. **(H)** Geometric mean of SIRPγ or **(I)** CD47 on Tregs are plotted. Each symbol represents independent HV. Statistical analysis was performed by a Friedman test followed by a Dunn’s multiple comparisons test. **(J)** A representative histogram of T effector (Teff) and T regulatory (Treg) cell proliferation under control conditions is shown at Day 5. **(K)** The effect of SIRPγ blockade with KWAR23 (10 µg/ml) was evaluated on T-cell proliferation upon OKT3 and anti-CD28 stimulation (both at 1 µg/ml). *n* = 2 independent experiments. **(L)** The consequences of SIRPγ blockade on Treg suppressive function was assessed by coculturing _ef450_CPD-labeled Teff with _ef670_CPD-labeled Treg cells in the presence or not of stimulatory molecule (OKT3) with or without SIRPγ blockade (KWAR23). Percentage of CPD^low^ effector T cells (± SEM) is presented regarding culture conditions. *n* = 4 independent experiments. ^*^ *p* ≤ 0.05; ^**^ *p* ≤ 0.01; ^***^ *p* ≤ 0.001; ^****^ *p* ≤ 0.0001; ns, not significant.

SIRPγ is expressed on T cells (16), and this expression can be altered by the presence of an eQTL (rs2281808) (20). So far, SIRPγ expression on different T-cell subsets has not been described. Thus, we analyzed SIRPγ expression on PBMC by FACS analysis and showed that naïve (CD3^+^CD45RA^+^CD28^+^) and central memory (CM, CD3^+^CD45RA^−^CD28^+^) T cells highly express SIRPγ whereas effector memory (EM, CD3^+^CD45RA^−^CD28^−^) T cells express to a lesser extent SIRPγ on their surface. Interestingly, TEMRA CD8 T cells (TEMRA, CD3^+^CD8^+^CD45RA^+^CD28^−^) express SIRPγ at a lower level than EM T cells (**Figures 2A, B** **)**. Since SIRPγ ligates CD47, a ubiquitously expressed molecule, we investigated CD47 expression on T-cell subsets. We show that it follows the same expression pattern as SIRPγ (**Figures 2A, C** **)**. We then evaluated SIRPγ expression on T cells upon polyclonal stimulation. Proliferating T cells rapidly and significantly upregulates SIRPγ at their surface and reaches a plateau once T cells have divided more than twice (**Figures 2D, E** **)**. This upregulation of SIRPγ is increasing from Days 3 to 5 of activation. Conversely, the expression of CD47 on proliferating T cells decreases upon T-cell divisions with a higher impact at Day 5 as compared with Day 3 (**Figures 2D, F** **)**.

We then investigated the expression of SIRPγ and CD47 on Tregs by gating on CD3^+^CD4^+^CD25^+^CD127^low^ cells and demonstrated that both molecules are highly expressed on naïve (CD45RA^+^) and memory/activated (CD45RA^−^) Tregs while CD47 is significantly less expressed by memory/activated Tregs (**Figures 2G** **–I**). Prior to questioning the role of SIRPγ on Treg functions, we evaluated the consequences of SIRPγ blockade on T-cell proliferation upon CD3 and CD28 stimulations. As shown in **Figure 2K**, the specific blockade of SIRPγ by KWAR23 slightly reduces T-cell proliferation. We have previously shown that CD28 stimulation can inhibit Treg function (21), thus in an experimental set-up where T effector cells were only stimulated with coated OKT3, we analyzed the impact of SIRPγ blockade on Tregs suppressive function. After 5 days of culture in anti-CD3-coated wells, effector T cells (Teff) proliferated, while Tregs cultured in the same conditions did not (**Figure 2J**). When cocultured at a 1:1 Teff : Treg ratio, Tregs were able to inhibit Teff proliferation regardless of the presence or not of KWAR23 (**Figure 2L**), suggesting that SIRPγ is not involved in Tregs suppressive function, although the direct effect of KWAR23 on OKT3-stimulated Teff and T reg cell would fully comfort our observations.

We further investigated the expression of SIRPγ on other cell types and detected it on MAIT cells, NKT cells (**Supplementary Figures S1A** **–C**), and in more than 60% of ILC1 cells (**Supplementary Figures S1D, E** **)**. SIRPγ was not expressed on B cells neither on NK cells (data not shown) as already reported by Piccio (16).

Altogether, our results show that SIRPγ expression varies upon T-cell differentiation and T-cell stimulation, suggesting that this molecule could impact T-cell functions.

### SIRPγ and CD47 Do Not Colocalize on Jurkat, Neither on Resting nor on Proliferating T Cells

**Figure 3:**
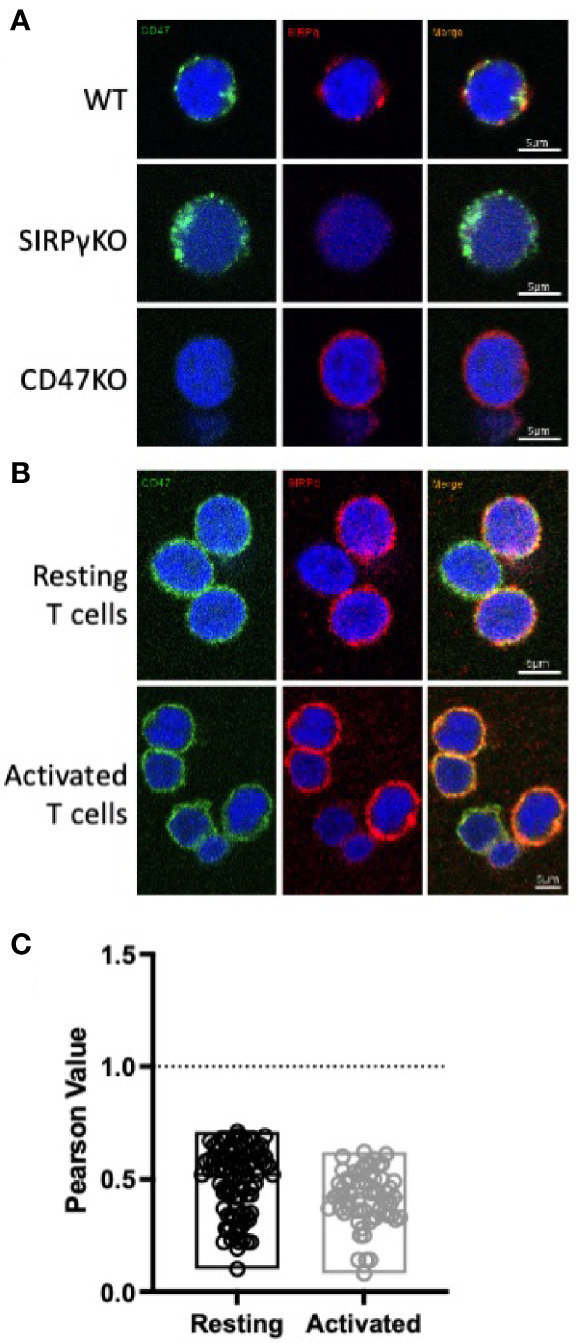
CD47 and SIRPγ do not colocalize on T cells. **(A, B)** Confocal microscopy imaging showing SIRPγ (red) and CD47 (green) cell surface distribution on Jurkat T **(A)**, freshly isolated T cells (**B**, upper panel) and activated T cells [**(B)**, lower panel]. Nuclei are stained with DAPI (blue). Images were taken with ×60 and ×2 zoom. **(C)** Colocalization of SIRPγ and CD47 on resting *vs*. activated T cells was investigated using ImageJ software and coloc2 plugin. Pearson value comprising between 0 (no colocalization) and 1 (colocalization) is presented for resting and activated T cells. *n* = 2 independent experiments.

Since SIRPγ and CD47 are both expressed on T cells with a similar expression pattern on the subpopulations, we hypothesized that CD47 may sequester SIRPγ at the cell surface by *cis* ligation on resting T cells. We first addressed this question using confocal microscopy by analyzing SIRPγ and CD47 expression patterns on wild-type (WT) Jurkat cells and on CRISPR-CAS9-modified Jurkat cells, i.e., Jurkat SIRPγ KO and Jurkat CD47 KO. On WT Jurkat cells, SIRPγ clusters in big punctuate patterns, whereas CD47 is expressed as smaller ones with a broad localization (**Figure 3A**); SIRPγ and CD47 therefore do not seem to colocalized on Jurkat cells. In the absence of SIRPγ, i.e., on SIRPγ KO Jurkat cells, CD47 expression is not altered and seems equivalent to that observed on WT Jurkat cells (**Figure 3A**). Surprisingly, the knockout of CD47 modifies the patterns of SIRPγ. SIRPγ is expressed on the whole cell surface instead of being in a big punctuate pattern at a specific localization of the cell surface as in WT Jurkat cells (**Figure 3A**). This might suggest that CD47 would impact SIRPγ distribution on the plasma membrane but not reciprocally. We also evaluated the expression of SIRPγ and CD47 on freshly isolated T cells; we identified a similar expression as on WT without any colocalization (**Figures 3B, C** **)**. Given that SIRPγ and CD47 expression varies upon T-cell stimulation (**Figures 2D** **–F**), we analyzed their distribution on resting and on activated T cells. Upon T-cell stimulation, SIRPγ is upregulated and expressed on the whole T-cell surface as compared with resting T cells (i.e., freshly isolated T cells), in which SIRPγ is localized in big punctuate patterns (**Figure 3B**). However, CD47 distribution does not change upon T-cell activation (**Figure 3B**). The Pearson value clearly showed that there is no colocalization of SIRPγ and CD47 neither at steady state nor upon T-cell activation since the value obtained were not close to 1 (**Figure 3C**).

### SIRPγ Polarizes at the Immune Synapse

**Figure 4:**
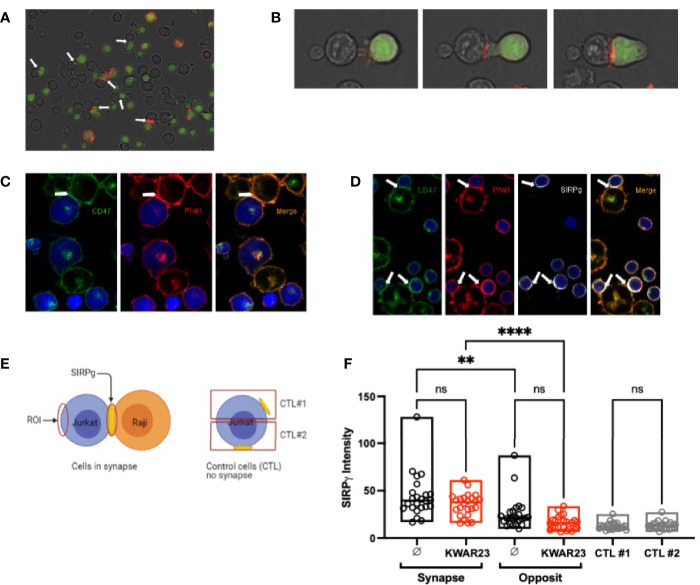
SIRPγ polarizes at the immune synapse. **(A)** Time-lapse microscopy of the polarization of SIRPγ at the Raji-Jurkat cell contact. Jurkat cells were stained with the cell tracker (green) and an anti-SIRPγ (red), and Raji cells (not stained) were primed with SEE; white arrows indicate SIRPγ clusters at the Jurkat-Raji cell contact. **(B)** Similar culture condition as in **(A)** with several screenshots from a time-lapse video highlighting SIRPγ cluster at the Jurkat-Raji cell contact. **(C)** SEE-primed Raji cells were stained with an anti-CD47 mAb (green). After the interaction with Jurkat cells (blue), cells were fixed, permeabilized, and stained with phalloidin (red) which shows a polarization of actine at the Raji-Jurkat cell contact, indicating a synapse formation. Green staining also shows a polarization of the CD47 of Raji at the synapse. **(D)** Similar culture condition as in **(A)** but with T cells instead of Jurkat cells. T cells (blue) were stained with anti-SIRPγ mAb (white), and Raji cells were stained with anti-CD47 mAb (green). After the interaction, cells were fixed, permeabilized, and stained with phalloidin (red). Phalloidin staining shows the polarization of actine in the immune synapse as well as SIRPγ and CD47. **(E)** Description of the methodology is used to analyze the expression of SIRPγ regarding cell situation. **(F)** Histograms showing SIRPγ intensity on Jurkat cells interacting or not (control (CTL)) with SEE-primed Raji cells in the presence or not of KWAR23. *n* = 5 to 10 cells analyzed per group from three independent experiments. Statistical analysis was performed by a Mann-Whitney test; ^**^ *p* ≤ 0.01; ^****^ *p* ≤ 0.0001, ns, not significant.

SIRPγ/CD47 interaction has been shown to enhance superantigen-mediated T-cell costimulation (16); we therefore hypothesized that SIRPγ might be localized at the immune synapse. Interestingly, we observed a polarization of SIRPγ by time-lapse microscopy when Jurkat and SEE-primed Raji cells are in close contact. Jurkat cells emit pseudopods when they are closed to Raji cells and SIRPγ clearly polarized at the cell-cell contact (**Figures 4A** **, B**; **Supplementary Video S1**). To ensure that this cell-cell contact was a synapse, in one experiment, cells were fixed, permeabilized, and stained with phalloidin. The staining showed a polarization of actine at the cell-cell contact indicating the synapse formation (**Figure 4C**). Interestingly, CD47 (stained only on Raji cells) was localized at the immune synapse (**Figure 4C**). We obtained similar results when using T cells (stained with an anti-SIRPγ Ab) from healthy volunteer (HV) rather than Jurkat (**Figure 4D**). We further quantified SIRPγ expression intensity at the synapse and on the opposite site of the cell as explained in **Figure 4E** in all acquired time-lapse microscopy videos and showed a clear SIRPγ accumulation at the immune synapse (**Figure 4F**). Interestingly, blocking the SIRPα-γ/CD47 interactions with KWAR23 mAb did not affect SIRPγ polarization at the synapse (**Figure 4F**), suggesting that SIRPγ spatial reorganization of the synapse does not depend on its interaction with CD47 on the Raji cells.

### Blocking SIRPγ/CD47 Interaction Impairs IFN-γ Secretion in Chronically Activated T Cells

**Figure 5:**
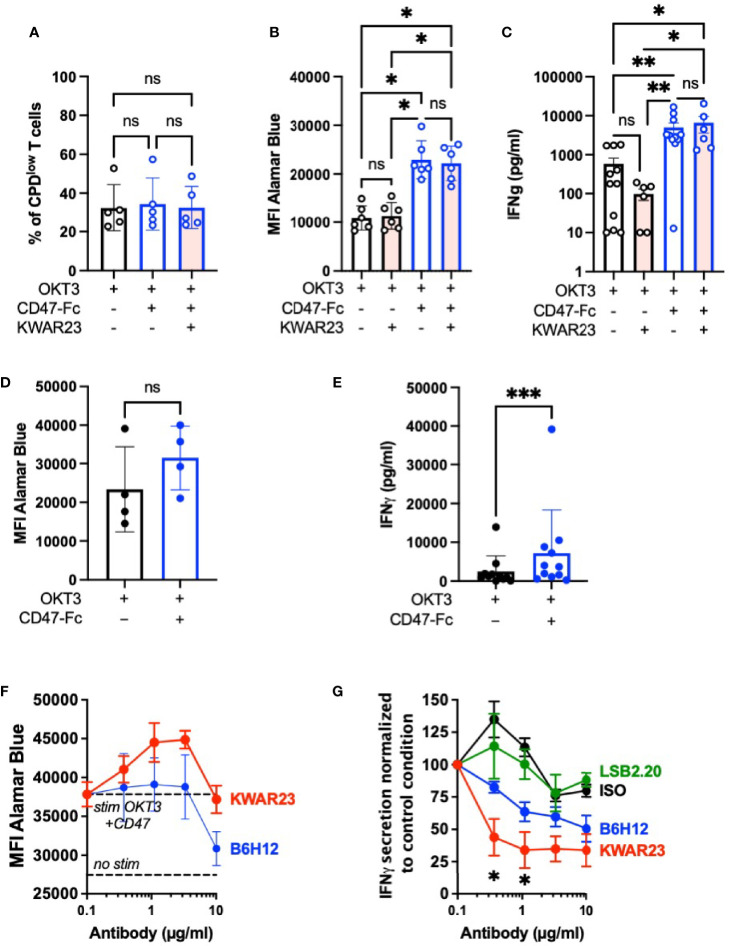
Inhibition of SIRPγ/CD47 interaction reduces IFN-γ secretion by chronically activated T cells. **(A**–**C)** T cells were stimulated once with coated OKT3 (0.5–1 µg/ml) with or without coated CD47-Fc (10 µg/ml) in the presence or not of KWAR23. **(A)** T-cell proliferation after 5 days of stimulation was analyzed by CPD dilution assay (*n* = 3 independent experiments with a total of four different HV). **(B)** T-cell fitness was evaluated with Alamar Blue at Day 5; *n* = 4. **(C)** IFN-γ dosage in the supernatant of T cells after 2 days of stimulation in depicted stimulation conditions (*n* = 12 within seven independent experiments). **(A**–**C)** Statistical analyses were performed with a one-way ANOVA test followed by Kruskal-Wallis; ^*^ *p* ≤ 0.05; ^**^ *p* ≤ 0.01; ^***^ *p* ≤ 0.001, ns, not significant. **(D, E)** T cell subjected to three rounds of stimulation with coated OKT3 and anti-CD28 were further stimulated by a coated OKT3 stimulation with or without coated CD47-Fc (10 µg/ml). **(D)** T-cell fitness was evaluated with Alamar Blue at Day 5. **(E)** Supernatant were collected 48 h after the last stimulation and IFN-γ secretion was evaluated by ELISA. **(D, E)** Statistical analyses were performed by a Wilcoxon test. ns, not significant. **(F, G)** T cells subjected to three rounds of stimulation as previously mentioned were further stimulated during 48 h by a coated OKT3 stimulation with or without coated CD47-Fc (10 µg/ml) with various concentrations of KWAR23, B6H12, or LSB2.2O. T-cell fitness was evaluated with Alamar Blue, *n* = 2 (mean ± SEM). **(G)** Supernatants collected at 48 h were analyzed by ELISA. Statistical analyses were performed by a Kruskal-Wallis test. ^*^ *p* ≤ 0.05 between KWAR23 and ISO and KWAR23 and LSB2.20 when Ab were used at 1.1 µg/ml; ^*^ *p* ≤ 0.05 between KWAR23 and ISO when Ab were used at 0.37 µg/ml. *n* = 3–5 (mean ± SEM).

To further decipher the importance of the SIRPγ/CD47 interaction on T-cell function, we stimulated T cells with low doses of OKT3 (0.5 or 1 µg/ml) and CD47-Fc, both coated, to provide a costimulatory signal as shown by Piccio and colleagues (16). With an experiment setup different than the one used by Piccio, we showed that the addition of coated CD47-Ig during T-cell stimulation did not increase their proliferation (**Figure 5A**); however, coated CD47-Ig increases T-cell metabolism independently of its interaction with SIRPγ since the addition of KWAR23 has no impact (**Figure 5B**). Importantly, coated CD47-Fc significantly enhances IFN-γ secretion of OKT3-stimulated T cells; once again the addition of KWAR23 in the culture has no effect (**Figure 5C**). Our results show that T cells freshly stimulated with coated OKT3 and coated CD47-Fc benefited from CD47 in a SIRPγ-independent pathway.

CD47 is upregulated by different cells upon chronic infection, notably viral infection (22), and tumor cells. We therefore evaluated the impact of CD47-Fc stimulation on T cells chronically stimulated with three rounds of anti-CD3 anti-CD28 stimulations. Chronically activated T cells further stimulated with CD47-Fc kept their fitness as shown in the **Figure 5D**. However, their culture on a OKT3-CD47 coating increased their IFN-γ secretion (**Figure 5E**). To evaluate the role of the SIRPγ/CD47 interaction in this observation, the blocking anti-SIRPγ antibody KWAR23 or the blocking anti-CD47 antibody B6H12 were respectively added. As shown in **Figure 5F**, the addition of KWAR23 did not impair cell fitness, while B6H12 seriously impaired T-cell viability when used at 10 µg/ml (**Figure 5F**). This mAb has already been shown to induce T-cell apoptosis (23). KWAR23 significantly decreased IFN-γ secretion while a tendency was observed with the blocking anti-CD47 antibody B6H12 (**Figure 5G**). This observation suggests a role of the SIRPγ/CD47 interaction in chronically activated T cells.

### Blocking SIRPα-γ/CD47 Interactions *In Vivo* Impairs Human T-Cell Chimerism and Delays the Xeno-GvHD Onset

**Figure 6:**
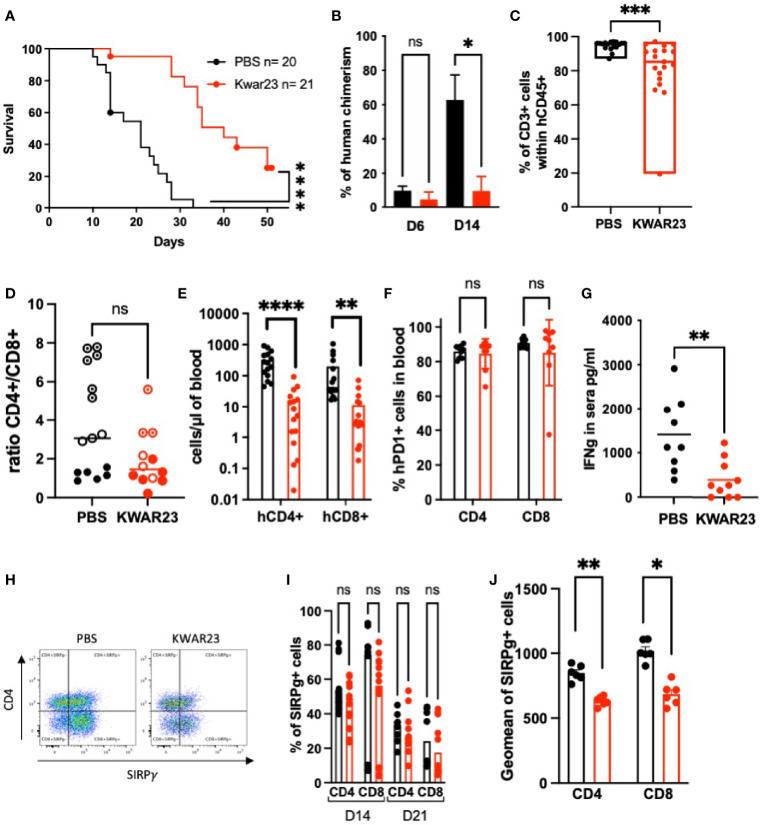
Inhibition of SIRPα-γ/CD47 interactions *in vivo* impairs human chimerism and delays xeno-GvHD. Recipient mice, irradiated at 1.5 Gy on Day 0, were injected i.v. with 10 million human PBMC 8 h later. Mice were i.p. treated 2×/week with the anti-SIRPα-γ mAb (KWAR23) at 5 mg/kg or with PBS until they reached notable clinical signs of xeno-GvHD. **(A)** Mice survival is shown regarding treatment. Data were analyzed with a Log-rank (Mantel-Cox) test. **(B)** Kinetics of human chimerism. Data were analyzed with a one-way ANOVA test followed by Kruskal-Wallis. **(C)** Percentage of human T cells within the human CD45+ population analyzed in the blood at D14; PBS-treated mice are presented in black, and KWAR23-treated mice are presented in red. Data were analyzed with a Mann-Whitney test. **(A**–**C)** Results from four experiments are presented in which human PBMC from four different healthy volunteers were injected in four to five mice per group. **(D)** CD4/CD8 ratio (percentage of positive cells within hCD45 population) was analyzed at D14 regarding the treatment. Data were analyzed with a Mann-Whitney test. *n* = 3 experiments with PBMC from three different HV donors (shown in different shape). **(E)** Absolute number of human CD4 and CD8 in the blood of recipient mice at Day 14. *n* = 3 experiments with PBMC from three different HV. Data were analyzed with a one-way ANOVA test followed by Kruskal-Wallis. **(F)** PD1^+^ cells within the circulating human CD4 and CD8 T cells; data were analyzed with a one-way ANOVA test. **(G)** IFN-γ dosage sera collected at Day 14 are shown; data were analyzed with a Mann-Whitney test. **(H)** Representative dot plots of human CD45^+^CD3^+^ in blood of humanized mice treated with PBS or KWAR23; SIRPγ expression on CD4^+^ and CD8 T cells (i.e., CD4^−^) is presented. **(I)** Percentage of SIRPγ^+^CD4^+^ and SIRPγ^+^CD8^+^ cells are shown and were analyzed with a one-way ANOVA test. **(J)** SIRPγ geometric mean on human CD4 and CD8 T cells. Data were analyzed with a one-way ANOVA test followed by Kruskal-Wallis. ^*^ *p* ≤ 0.05; ^**^ *p* ≤ 0.01; ^***^ *p* ≤ 0.001; ^****^ *p* ≤ 0.0001; ns, not significant.

Since SIRPγ is absent in rodents, we took advantage of a graft-*versus*-host disease (GvHD) model, in which human PBMC injected in NSG-recipient mice would be chronically stimulated by the xenogeneic environment. The role of SIRPγ in this experimental condition was evaluated by treating or not the irradiated recipient mice with 5 mg/kg of KWAR23 twice a week. In this situation, the xenogenic GvHD is human T cells driven due to their cross-reactivity with host MHCs (24). We first observed that mice survival was significantly extended when treated with KWAR23 (**Figure 6A**). At Day 6, mice treated with the KWAR23 showed a tendency of a lower human chimerism, although the frequency of human cells within the blood was quite low 1-week posthumanization. This observation was clearly significant at Day 14 (**Figure 6B**), suggesting that SIRPγ blockade impairs T-cell survival, activation, proliferation, or localization within the host. This chimerism mostly comprised human CD3^+^ cells in both groups as shown by blood phenotypic analysis at Day 14 (**Figure 6C**). Human CD8 T cells played a primary role in xeno-GvHD pathogenicity (24); however, the CD4/CD8 ratio analyzed at D14 did not show an accumulation of CD8 T cells in the blood (**Figure 6D**). At Day 14, lower absolute numbers of human CD4 and CD8 circulating T cells were detected in the KWAR23-treated group (**Figure 6E**) which could explain the delay of the observed xeno-GvHD onset. Mice with a NOD background such as NSG mice, are superior to other strains for human immune cell engraftment thanks to their SIRPα whose affinity for human CD47 is even higher than the one for their natural ligand (mCD47), ensuring a functional “don’t eat me*”* signal (25). NSG mice are known to have functional residual macrophages. Given the weak chimerism observed in the KWAR23-treated group and the specificity of the KWAR23 to block human SIRPα-γ/hCD47 interactions, we wondered whether the KWAR23 cross-reacted with mouse SIRPα leading to the disruption of mSIRPα/hCD47 *don’t eat me* signal and eventually the phagocytosis of human cells. Intraperitoneal macrophages collected from NSG mouse are all CD11b^+^ SIRPα^+^ and are not stained by the KWAR23 (**Supplementary Figure S2A**), ruling out our previously depicted hypothesis.

We evaluated the consequences of the KWAR23 treatment on T-cell activation. We analyzed PD1 expression on human circulating cells. All human T cells were PD1^+^ regardless of the treatment (**Figure 6F**). Therefore, the delay of the GvHD onset in the KWAR23-treated group was not due to a defect of human T-cell activation. *In vitro*, we have shown that chronically activated T cells secreted less IFN-γ when SIRPγ/CD47 interaction was blocked (**Figure 5G**). Thus, we measured IFN-γ concentration in mice’s sera collected at Day14. As expected, KWAR23-treated mice secreted significantly less IFN-γ than control mice (**Figure 6G**). This can be explained either by the lower amount of human T cells within the KWAR23-treated group, or by a direct effect of the treatment on chronically activated T cells (as shown *in vitro* in **Figure 5G**). Because human T cells are fully activated in the xenogeneic environment, we wondered if the KWAR23 treatment would impact the CD47’s downmodulation observed *in vitro* following T-cell stimulation (**Figure 2F**). Indeed, the downmodulation of human CD47 might reduce the “*don’t eat me*” signal and increase their susceptibility to residual mouse macrophages. KWAR23 did not stress CD47 down-regulation (**Supplementary Figure S2B**) indicating that the treatment would not increase T-cell susceptibility to mouse macrophages.

We then evaluated SIRPγ on human T cells in both groups by using the anti-SIRPγ mAb clone LSB2.20. We first validated that the KWAR23 did not impair the staining using LSB2.20 (**Supplementary Figure S2C**). The frequency of human CD4 and CD8 SIRPγ^+^ T cells in the KWAR23-treated group was not different to that in the PBS-treated mice (**Figures 6H, I** **)**. However, SIRPγ geometric mean was significantly lower in the KWAR23-treated group (**Figures 6H** **–J**).

Thus, blocking SIRPγ/CD47 interaction *in vivo* impaired human T-cell chimerism by specifically affecting human T-cell numbers leading to the delay of xeno-GvHD.

### Blocking SIRPα-γ/CD47 Interactions *In Vivo* Impair Human T-Cell Differentiation

**Figure 7:**
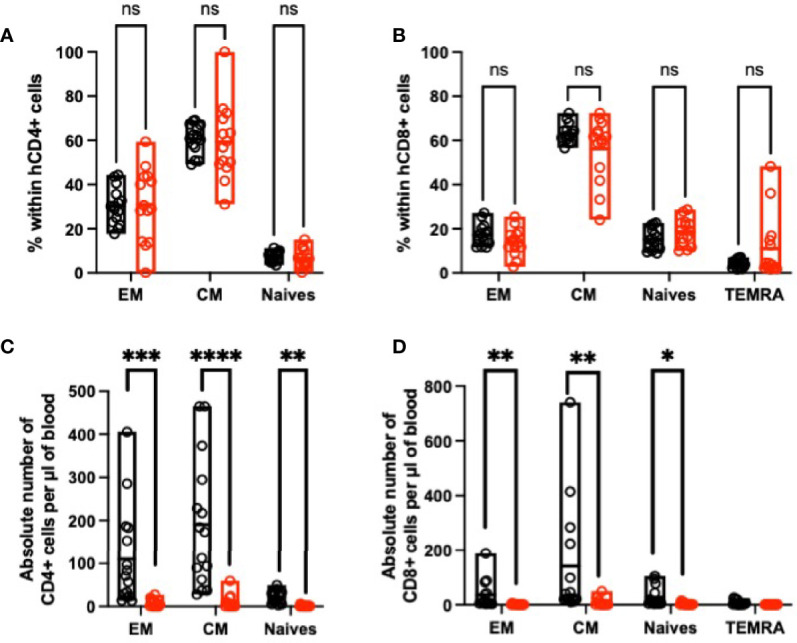
Inhibition of SIRPα-γ/CD47 interactions alters blood memory cells in humanized mice. At Day 14, blood cells were analyzed by FACS. **(A)** Percentage of human CD4 and **(B)** CD8 subpopulations are presented based on FACS analysis with the following patterns CD45RA^−^CD27^−^ effector memory cells (EM), CD45RA^−^CD27^+^ central memory cells (CM), CD45RA^+^CD27^+^-naïve cells, and CD4^−^CD45RA^+^CD27^−^ for TEMRA CD8 cells. **(C)** Absolute number of each subpopulation of CD4^+^ and **(D)** CD8^+^ T cells are presented. *n* = 3 experiments with PBMC from three different HV. Data were analyzed with a one-way ANOVA test followed by Kruskal-Wallis. ^*^ *p* ≤ 0.05; ^**^ *p* ≤ 0.01; ^***^ *p* ≤ 0.001; ^****^ *p* ≤ 0.0001; ns, not significant.

**Figure 8:**
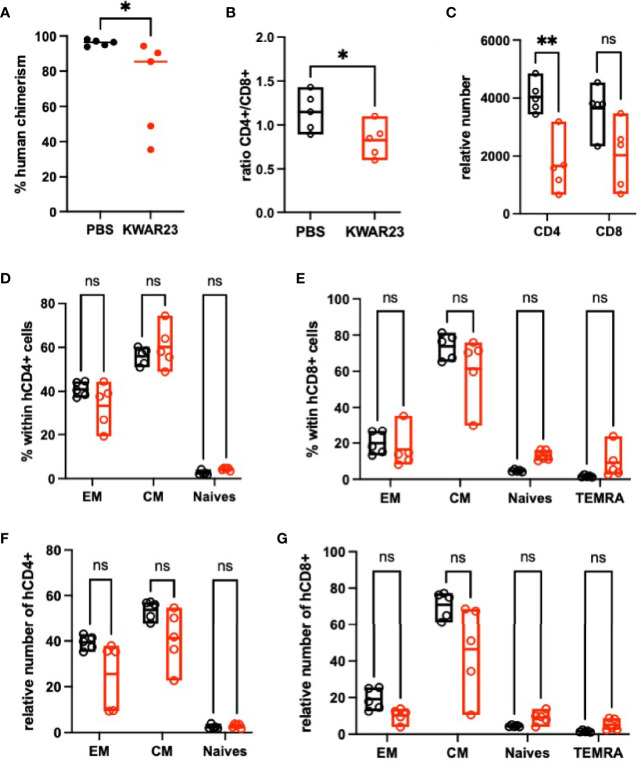
Inhibition of SIRPα-γ/CD47 interactions alters memory cells in the spleen of humanized mice. At Day 14, splenocytes were analyzed by FACS. **(A)** Human chimerism is shown; data were analyzed with a Mann-Whitney test. **(B)** CD4/CD8 ratio is shown regarding the treatment; data were analyzed with a Mann-Whitney test. **(C)** Relative number of human CD4 and CD8 in the splenocytes of recipient mice at Day 14. Data were analyzed with a one-way ANOVA test followed by Kruskal-Wallis. **(D)** Percentage of human CD4 and **(E)** CD8 subpopulations is presented based on FACS analysis with the following patterns CD45RA^−^CD27^−^ effector memory cells (EM), CD45RA^−^CD27^+^ central memory cells (CM), CD45RA^+^CD27^+^-naïve cells, and CD4^−^CD45RA^+^CD27^−^ for TEMRA CD8 cells. **(F)** Relative number of each subpopulation of CD4^+^ and **(G)** CD8^+^ T cells are presented. *n* = 1 experiment with PBMC from one HV. Data were analyzed with a one-way ANOVA test followed by Kruskal-Wallis. ^*^ *p* ≤ 0.05; ^**^ *p* ≤ 0.01; ns, not significant.

Because SIRPγ expression varies upon T-cell differentiation in the blood from HV (**Figures 2A** **–C**), we evaluated the impact of the treatment on T-cell differentiation *in vivo*. Three out of four HV whose PBMC were i.v. injected were phenotyped prior to their injection in NSG mice; naïve CD4, central memory CD4, and central memory CD8 T cells were the most abundant subpopulations (**Supplementary Figures S3A, B** **)**. After i.v. hPBMC injection in untreated recipient mice, naïve T-cell compartment shrank in favor to central memory and effector memory cells regardless of the treatment. Whether or not this observation reflects naïve T-cell differentiation into central memory and effector memory cells, together with a memory T-cell compartment activation is difficult to evaluate. However, the inhibition of SIRPα-γ/CD47 interactions *in vivo* did not bias neither CD4 nor CD8 T-cell subpopulation frequencies (**Figures 7A, B** **)** while it clearly affected the absolute number of CD4 and CD8 T in all subpopulations except the TERMA CD8 T cells (**Figures 7C, D** **)**. To further document the effect of KWAR23-treatment, some mice were sacked and their splenocytes were analyzed at Day 14. We confirmed that human cell engraftment was impaired in the spleen also upon KWAR23 treatment (**Figure 8A**). The CD4/CD8 ratio was significantly lower in the KWAR23-treated group (**Figure 8B**). A relative number of CD4 was significantly lower in the KWAR23-treated group (**Figure 8C**) without affecting the frequency of CD4 or CD8 T-cell subpopulations (**Figures 8D, E** **)** nor the relative number of those subpopulations (**Figures 8F, G** **)**.

Altogether, our results suggest that KWAR23 treatment affects survival of all human T-cell subpopulations independently of mice macrophage implication.

## Discussion

SIRPγ protein is only expressed in humans and nonhuman primates (26) and binds CD47, an ubiquitous protein with an affinity 10-fold lower than that of SIRPα for CD47 (15). So far, reports on the potential role of the SIRPγ were performed with an anti-SIRPγ antibody (clone LSB2.20) which has not been appropriately characterized limiting the interpretation of previous reports on the SIRPγ biology. Thus, we decided to reevaluate the impact of SIRPγ in human T-cell biology using a different anti-SIRP monoclonal antibody (clone KWAR23) which we characterized here as capable to efficiently block SIRPγ and SIRPα interactions with CD47. Addressing this question by blocking at the CD47 protein as previously reported (16) is challenging since CD47 is a multifacetted target engaging with various ligands in *cis* and in *trans*-interaction (27, 28), and some anti-CD47 mAb known to block SIRP/CD47 interaction (clone B6H12) can also trigger T-cell apoptosis (23).

Here, we report that SIRPγ is expressed by all naïve and central memory T cells and is upregulated after activation as previously reported (15), confirming that T cells might benefit from this expression. While a previous study (16) reported that anti-SIRPγ (LSB2.20 clone) or anti-CD47 (clone B6H12) negatively impact human T-cell activation, we were first surprised that the specific blockade of SIRPγ/CD47 interaction using appropriate tools did not seem to directly impact T-cell activation upon one round of stimulation at least on T-cell functions addressed in our experiments, i.e., proliferation, metabolism, and IFN-γ secretion which are one of the many aspects of T-cell activation. The benefits of CD47-Ig coating on OKT3-stimulated T cells might therefore be dependent on a trans CD47/CD47 interaction as mentioned by others (27, 28).

SIRPγ extracellular portion consists of three domains (D1, D2, and D2), and isoforms containing D1/D2/D3, D1/D2, or D1 only, have been described (14, 16). SIRPγ/CD47 interaction was suggested to occur the SIRPγ D1 portion (29, 30). The LSB2.20 was raised against a SIRPγ-D1/D2-Ig fusion protein. Thus, one could consider that LSB2.20 epitope is not on the same extracellular domain than CD47-binding site. This would explain that targeting SIRPγ with either CD47-Ig or LSB2.20 on T cells would differently act on their effector functions as presented here and in a former study by Piccio for CD47-Ig or LSB2.20 stimulation, respectively (16). Recently, a patent by Ruozhen and colleagues in which epitopes of different anti-SIRPγ are mentioned, indicates that LBS2.20-binding domain is on SIRPγ D1 (31), so on the same domain than the CD47-binding site. Furthermore, they also showed LSB2.20 inefficient to increase T-cell proliferation upon CD3 stimulation (31).

Given the broad distribution of CD47, one could consider that its implication in T-cell biology through SIRPγ ligation should be finely tuned. Thus, we hypothesized that an upregulation of CD47 could impact SIRPγ ligation and lead to a biological effect. Indeed, CD47 is upregulated in a number of cancer cells, notably by *MYC* oncogene, a transcription factor, which binds promotor of *cd47* and *Pdl-1* genes (32). CD47 upregulation has been very recently shown in infected cells (22, 33). Here, we describe that SIRPγ clusters at the immune synapse and CD47 on the target cells also clusters at the cell/cell contact in a juxtacrine fashion. These observations confirm that SIRPγ expression and reorganization should also benefit human T cells. Unlike CD28 (34) on T cells or SIRPα on macrophages (35), SIRPγ localization at the immune synapse seem independent of its ligation to its known ligand CD47. This may reflect that SIRPγ/CD47 interaction needs to exceed a threshold for its effector functions, explaining SIRPγ localization to the immune synapse independently of CD47 engagement as a sensor searching for sufficient CD47 expression on the target cells.

It is worth noting that we could not reveal CD47 on WT Jurkat cells when using SIRPγ-Fc by FACS, while the SIRPα-Fc could. This may be due to the 10-fold lower affinity of SIRPγ, to the level of CD47 expressed by Jurkat cells or to the conformation of either CD47 or SIRPγ. pH may influence protein/protein interactions as acidic pH has just unrevealed VISTA as a new ligand of PSGL1 (36), given that CD47 is upregulated by tumor cells in a usually acidic environment, pH variation might also modify CD47/SIRPγ affinity.

In this study, all our experimental readouts focused on T-cell biology considering SIRPγ as a ligand. However, the 4 aa long SIRPγ intracellular tail is unexpected to transduce a signal by its own. Unlike SIRPβ, SIRPγ is not associated with DAP12 (4, 15, 16). Recently, the polymorphism of SIRPα (i.e., SIRPα-V1 and SIRPα-V2) has been shown to stimulate CD47 when donor differed from the recipient in one or both *Sirpa* alleles resulting in innate immune responses (37). Although these experiments were addressed in mice and not yet confirmed in humans, one can consider that upregulation of SIRPγ could contribute in a CD47 signaling.

Interestingly, all poxviruses that infect vertebrates (except four) contain predicted vCD47s (38). Even though their amino acid sequence similarity with their host parent gene is comprised between 18% and 29%, all of them conserved the structure with a N-terminal IgV domain, five transmembrane helices, and a short cytoplasmic tail (38). Although smallpox was recently eradicated by vaccination, the virus was present for thousands of years; SIRPγ may have interacted with vCD47. Recently, Myers and colleagues have shown that mice chronically infected with Friend retrovirus or LCMV Clone 13 accumulate some SIRPα^+^CD8^+^ T cells (39). They also evidenced this population in patients with chronic HCV infections by using a CyTOF approach. The expression of SIRPα was lower in these CD8 T cells than in monocytes. In mice, the SIRPα expression on T cells was associated with effector functions; in humans, SIRPα^+^CD8^+^ T cells show higher activation status as compared with their SIRPα^−^CD8^+^ counter parts (39). KWAR23, blocking anti-SIRPγ antibody, treatment was mostly effective in chronically activated T cells *in vitro* (**Figure 5**) and *in vivo* (**Figure 6A**). We previously reported that SIRPγ/CD47 blockade inhibits IFN-γ release in human tumor-antigen T-cell cross-priming assays using a CD8^+^ T-cell clone isolated and expanded from a melanoma patient (40, 41). Altogether, this suggests that SIRPγ/CD47 interaction might be more important in the activation biology of memory or chronically activated human T cells, than in naïve T-cell costimulation as previously hypothesized. Moreover, SIRPγ/CD47 interaction could play other roles, such as positively control the human T-cell transendothelial migration under shear flow conditions (17) or tissue migration (40). Since KWAR23 antibody blocks both SIRPα and SIRPγ interactions with CD47, we might consider that the observed effects were not only due to the sole SIRPγ blockade but also to SIRPα/CD47 blockade. However, on one hand *in vivo*, this antibody displays no cross-reactivity for mouse SIRPα and on the other hand *in vitro* in our chronically activated human T-cell models, only T cells remained after several polyclonal stimulation rounds.

Finally, we were surprised that the SIRPγ spatial reorganization at the synapse did not depend on its interaction with CD47. As previously mentioned, SIRPγ affinity for CD47 is relatively low and CD47 homophilic interactions have already been shown (27) which could explain CD47 clustering at the Raji membrane, although CD47KO target cells (here Raji) would be needed to fully confirm our observation. SIRPγ might interact with a yet unknown other receptor (beside CD47) which interaction might not be blocked by the KWAR23 antibody.

In the study, using a blocking anti-SIRPα-γ mAb, we report that T-cell interaction with CD47 is of importance not only for T-cell migration but also for their activation particularly in chronic stimulation situations. Whether these observations only involve SIRPγ or SIRPγ and SIRPα proteins remain to be clarified.

## Data Availability Statement

The original contributions presented in the study are included in the article/**Supplementary Material**. Further inquiries can be directed to the corresponding author.

## Ethics Statement

Blood from healthy individuals was obtained at the Etablissement Français du Sang (Nantes, France). Written informed consent was provided according to institutional guidelines. The patients/participants provided their written informed consent to participate in this study. This study was carried out according to authorization from the French Ministry of Research, APAFIS # 4678.

## Author Contributions

SD, VN-D, MN, NE-D, J-MH, LB, CM, VT, and KB performed the experiments. SD, VN-D, NE-D, LB, and CM analyzed the experiments. NP, GB, and FH designed the experiment and overviewed the study. GB contributed to the writing of the paper. NP and GB obtained funding for the project. SD, NP, and FH wrote the paper. All authors contributed to the article and approved the submitted version.

## Funding

This work was supported by the French Public Bank of Investment (BPI EFFI-CLIN PSPC grant) and the Humanized Rodent Platform was supported by the Labex IGO project (ANR-11-LABX-0016-01) funded by the «Investissements d’Avenir» French Government program, managed by the French National Research Agency (ANR) (http://www.labex-igo.com/).

## Conflict of Interest

SD, LB, CM, VT, KB, and NP are employees and shareholders of OSE Immunotherapeutics, a company developing SIRPα antagonists. CM, VT, and NP are authors of patents related to SIRPγ antagonists.

The remaining authors declare that the research was conducted in the absence of any commercial or financial relationships that could be construed as a potential conflict of interest.

## Publisher’s Note

All claims expressed in this article are solely those of the authors and do not necessarily represent those of their affiliated organizations, or those of the publisher, the editors and the reviewers. Any product that may be evaluated in this article, or claim that may be made by its manufacturer, is not guaranteed or endorsed by the publisher.

## Supplementary Material

Supplementary Figure S1 Expression of SIRPγ on blood cells other than T cells. SIRPγ expression has been analyzed on blood immune cells other than T cells. **(A)** Dot plot of SIRPγ expression on MAIT (CD45^+^CD3^+^TCRαβ^+^CD161^+^Vα7.2^+^ cells) and NKT (CD45^+^CD3^+^ CD56^+^) cells. **(B)** Geometric mean of SIRPγ on MAIT and NKT cells. Each symbol represents a HV. **(C)** Percentage of MAIT and NKT cell-expressing SIRPγ. **(D)** SIRPγ on ILCs (CD45^+^Lin^−^CD127^+^) is presented as dot plots; NCR marker, CRTH2, and CD161 were used to analyze the subsets of ILC. **(E)** Percentage of ILC-expressing SIRPγ, *n* = 3 independent HV.

Supplementary Figure S2 The KWAR23 treatment does not affect the analysis of human cells *in vivo*. **(A)** Intraperitoneal NSG cells were collected and stained for mouse SIRPα and mouse CD11b. The cross reactivity of the KWAR23 (antihuman SIRPα-γ) against mSIRPα was evaluated. CD11b and SIRPα^+^ expression on intraperitoneal macrophages is presented as dot plot and the cross reactivity of the KWAR23 (revealed with the antihuman IgG PE) is presented in the red histogram while the control staining with the antihuman IgG PE only is presented in the black histogram (upper panel). In the same experiment, human PBMC were used as control and were either stained with KWAR23 and revealed with the antihuman IgG PE (red histogram) or were only stained with the antihuman IgG PE (black histogram). **(B)** Geometric mean (± SEM) of CD47 expression on CPD-stained T cells is presented as a function of their division numbers, in the presence (red) or not (black) of KWAR23 during an anti-CD3+ anti-CD28.2 stimulation. *n* = 4 HV analyzed in one experiment. **(C)** SIRPγ staining with LSB2.20 on human CD3^+^ PBMC (left dot plot) and on human PBMC precoated with KWAR23 at 5 µg/ml during 25 min on ice (right dot plot).

Supplementary Figure S3 T-cell phenotype of human PBMC prior to their i.v. injection in NSG. PBMC from three HV were analyzed by FACS prior their i.v. injection in NSG-irradiated recipient mice. Percentage of CD4 **(A)** and CD8 **(B)** subpopulations are presented based on FACS analysis with the following patterns CD45RA^−^CD27^−^ effector memory cells (EM), CD45RA^−^CD27^+^ central memory cells (CM), CD45RA^+^CD27^+^-naïve cells, and CD4^−^CD45RA^+^CD27^−^ for TEMRA CD8 cells.

Supplementary Video S1 SIRPγ polarizes at the Jurkat-Raji immune synapse. Time-lapse microscopy of the polarization of SIRPγ at the Raji-Jurkat cell contact. Jurkat cells were stained with a cell tracker (green) and the anti-SIRPγ (red); Raji cells (not stained) were primed with SEE.
