# Supplementary figures and images for: SIRPγ-CD47 Interaction Positively Regulates the Activation of Human T Cells in Situation of Chronic Stimulation
Source: Front Immunol. 2021 Dec 1;12:732530. doi: 10.3389/fimmu.2021.732530 (PMC8671138; doi:10.3389/fimmu.2021.732530)

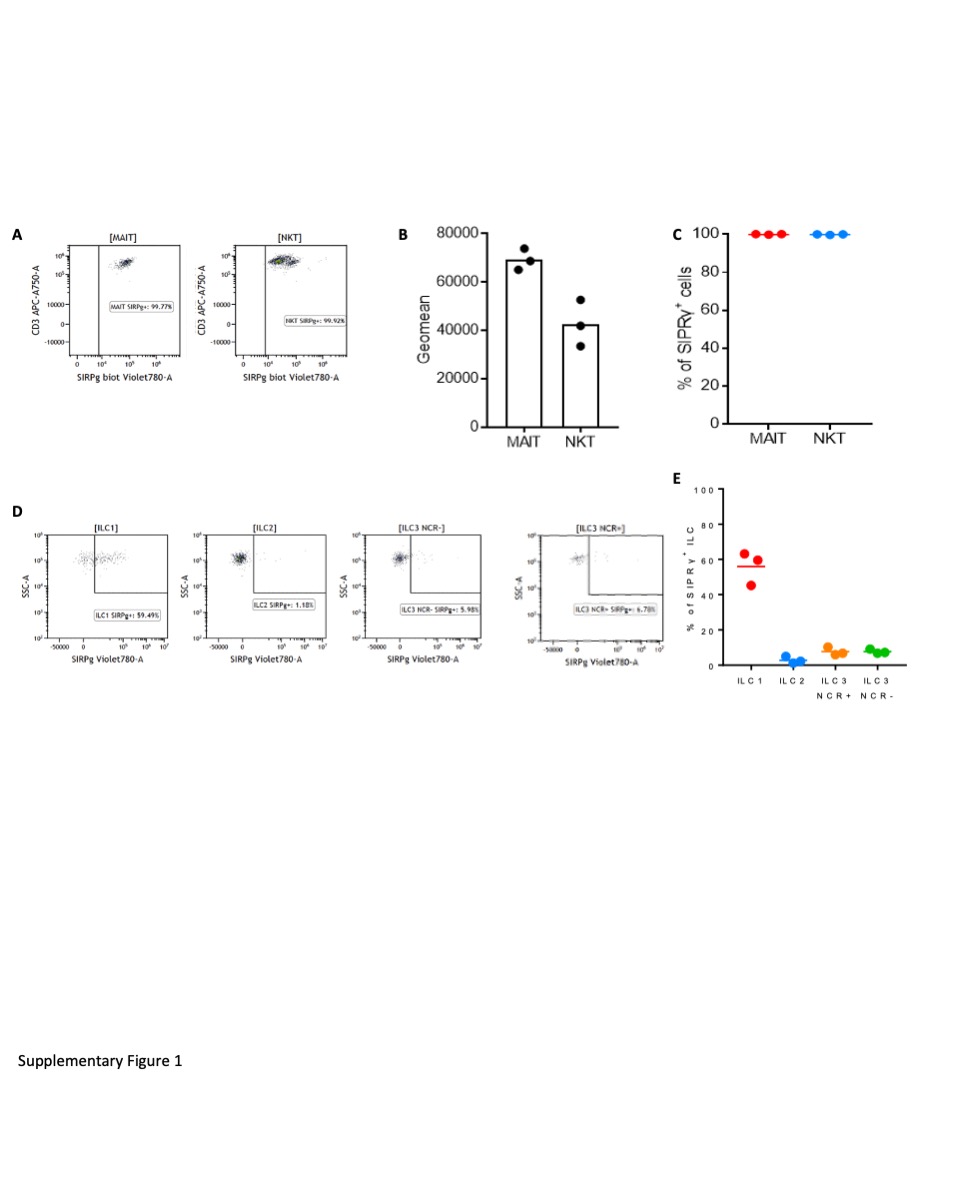

Supplement: Supplementary Figure S1 — Expression of SIRPγ on blood cells other than T cells. SIRPγ expression has been analyzed on blood immune cells other than T cells. (A) Dot plot of SIRPγ expression on MAIT (CD45+CD3+TCRαβ+CD161+Vα7.2+ cells) and NKT (CD45+CD3+ CD56+) cells. (B) Geometric mean of SIRPγ on MAIT and NKT cells. Each symbol represents a HV. (C) Percentage of MAIT and NKT cell-expressing SIRPγ. (D) SIRPγ on ILCs (CD45+Lin−CD127+) is presented as dot plots; NCR marker, CRTH2, and CD161 were used to analyze the subsets of ILC. (E) Percentage of ILC-expressing SIRPγ, n = 3 independent HV. [file Image_1.jpeg]

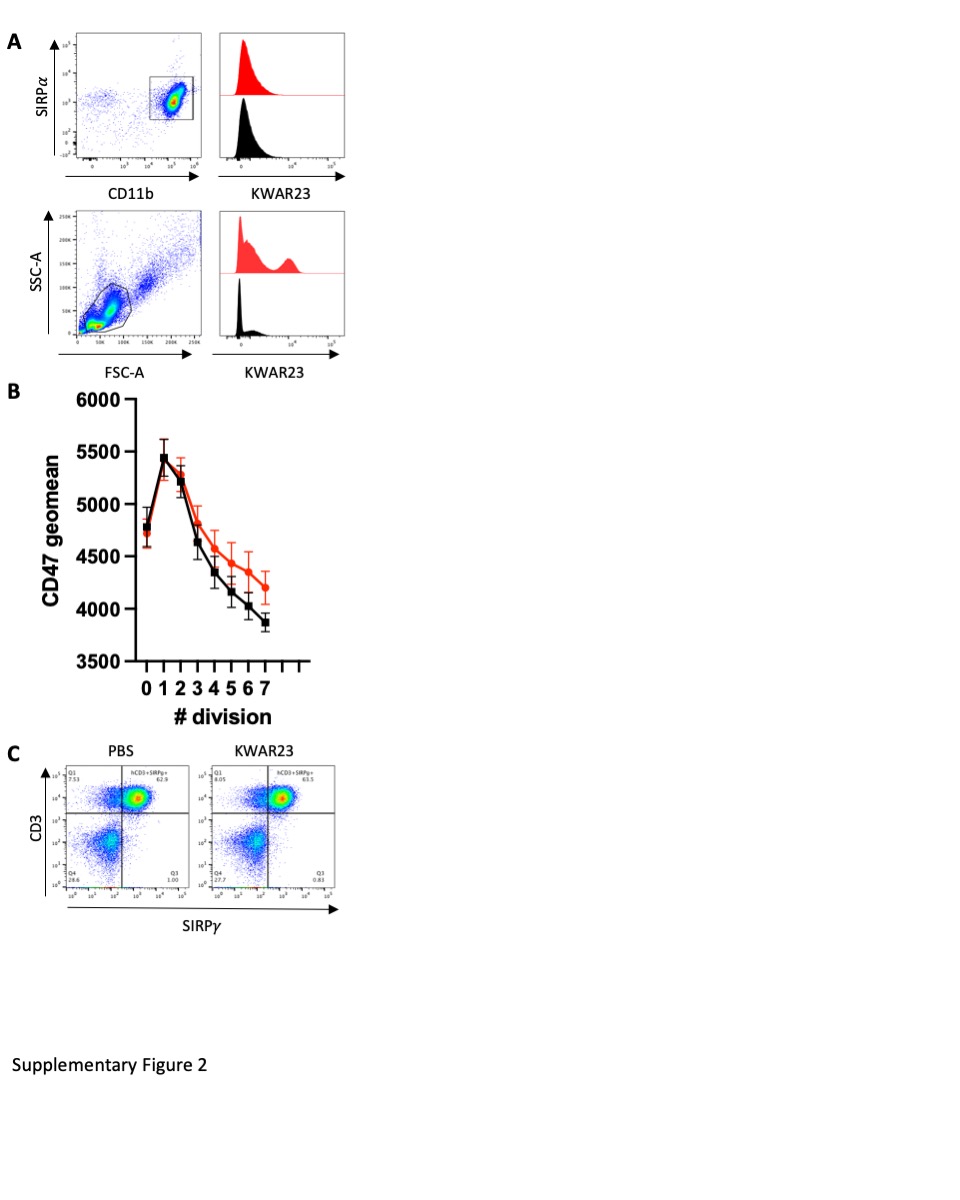

Supplement: Supplementary Figure S2 — The KWAR23 treatment does not affect the analysis of human cells in vivo. (A) Intraperitoneal NSG cells were collected and stained for mouse SIRPα and mouse CD11b. The cross reactivity of the KWAR23 (antihuman SIRPα-γ) against mSIRPα was evaluated. CD11b and SIRPα+ expression on intraperitoneal macrophages is presented as dot plot and the cross reactivity of the KWAR23 (revealed with the antihuman IgG PE) is presented in the red histogram while the control staining with the antihuman IgG PE only is presented in the black histogram (upper panel). In the same experiment, human PBMC were used as control and were either stained with KWAR23 and revealed with the antihuman IgG PE (red histogram) or were only stained with the antihuman IgG PE (black histogram). (B) Geometric mean (± SEM) of CD47 expression on CPD-stained T cells is presented as a function of their division numbers, in the presence (red) or not (black) of KWAR23 during an anti-CD3+ anti-CD28.2 stimulation. n = 4 HV analyzed in one experiment. (C) SIRPγ staining with LSB2.20 on human CD3+ PBMC (left dot plot) and on human PBMC precoated with KWAR23 at 5 µg/ml during 25 min on ice (right dot plot). [file Image_2.jpeg]

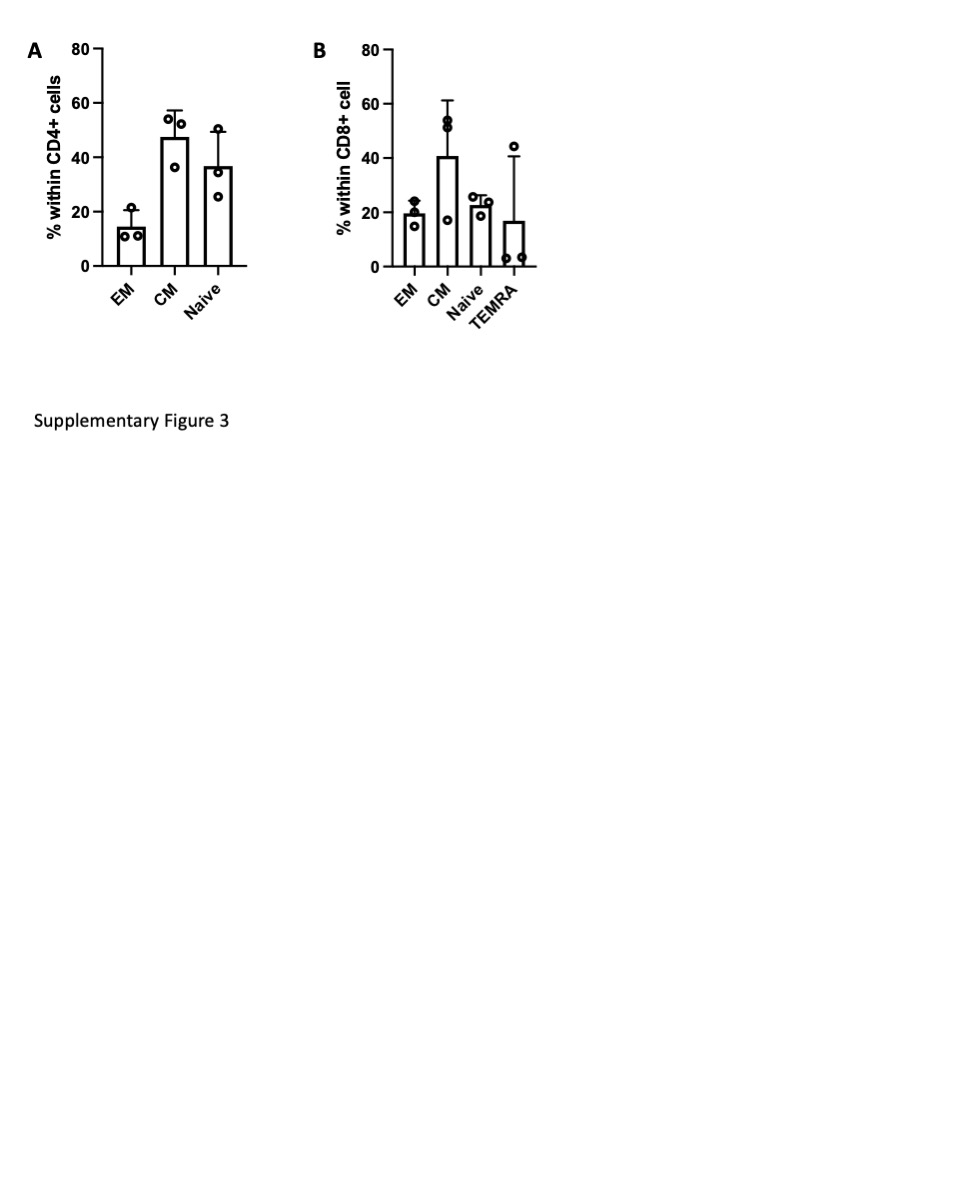

Supplement: Supplementary Figure S3 — T-cell phenotype of human PBMC prior to their i.v. injection in NSG. PBMC from three HV were analyzed by FACS prior their i.v. injection in NSG-irradiated recipient mice. Percentage of CD4 (A) and CD8 (B) subpopulations are presented based on FACS analysis with the following patterns CD45RA−CD27− effector memory cells (EM), CD45RA−CD27+ central memory cells (CM), CD45RA+CD27+-naïve cells, and CD4−CD45RA+CD27− for TEMRA CD8 cells. [file Image_3.jpeg]
